# HIN9/468: The Last Mile - Secure and mobile data processing in healthcare

**DOI:** 10.2196/jmir.1.suppl1.e40

**Published:** 1999-09-19

**Authors:** HB Bludau, A Vocke, W Herzog

**Affiliations:** ^1^Universität Heidelberg, HeidelbergGermany

**Keywords:** Mobile Computing, Technology Assessment, Clinical Information Systems

## Abstract

**Motivation:**

According to the Federal Ministry the avowed target of modern medicine is to administer the best medical care, the newest scientific insights and the knowledge of experienced specialists on affordable conditions to every patient no matter whether he is located in a rural area or in a teaching hospital. One way of administer information is on mobile tools. To find out more about the influence of mobile computer on the physician-patient relation, the acceptance of these tools as well as prerequisites of new security and data-processing concepts were investigated in a simulation study.

**Methods:**

The Personal Digital Assistant

Based on a personal digital assistant a prototype was developed. The Apple Newton was used because it appeared suitable for easy data input and retrieval by the means of a touch screen with handwriting recognition. The device was coupled with a conventional cellular phone for voice and data transfer The prototype provided several functions for information processing:

- access to a patient database
- access to medical knowledge
- documentation of diagnosis
- electronic requests forms for investigations
- tools for the personal organization.

The prototype of an accessibility and safety manager was integrated. This software enables to control telephone accessibility individually. Situational adjustments and a complex set of rules configured the way arriving calls were dealt with. Moreover this software contained a component for sending and receiving text messages.

**The Simulation Study**

In simulation studies, test users are observed while working with prototypical technology in a close-to-reality environment. The aim is to test an early prototype in its avowed environment to obtain design proposals for technology by the future users. Within the Ladenburger group "Security in communications technology" of the Gottlieb-Daimler und Karl-Benz-Stiftung an investigation at the Heidelberg University Medical Centre was conducted under organisational management of the project group "Verfassungsverträgliche Technikgestaltung (Provet)". Thirty-one health professionals tested the device described above. Data about the user experience were obtained by:

- observation while using the device
- evening meeting with main topic
- structured questionnaire
- individual interview

**Results:**

After the investigation week more than 2/3 of the participants indicated that they expect no or a positive modification of their communication behaviour. To emphasise is a group of test persons (one out of five), who feared a negative modification of their communication behaviour. Mobile data processing was judged as important and useful by more than two third of the professionals asked.

**Conclusions:**

At present the original concept of a multifunctional device with integration of mobile voice and data communication did not prove as practicable. Rather a multi-device concept with stationary, mobile and hand-held device has to be developed.

